# Vaccine effectiveness against emerging COVID-19 variants using digital health data

**DOI:** 10.1038/s43856-024-00508-9

**Published:** 2024-05-06

**Authors:** Tanner J. Varrelman, Benjamin Rader, Christopher Remmel, Gaurav Tuli, Aimee R. Han, Christina M. Astley, John S. Brownstein

**Affiliations:** 1 Computational Epidemiology Lab, Boston Children’s Hospital, Boston, MA 02115 USA; 2 Department of Epidemiology, Boston University, Boston, MA 02118 USA; 3 Division of Endocrinology, Boston Children’s Hospital, Boston, MA 02115 USA; 4 Harvard Medical School, Boston, MA 02115 USA; 5 Broad Institute of Harvard and MIT, Cambridge, MA 02142 USA

**Keywords:** Diseases, Computational biology and bioinformatics

## Abstract

**Background:**

Participatory surveillance of self-reported symptoms and vaccination status can be used to supplement traditional public health surveillance and provide insights into vaccine effectiveness and changes in the symptoms produced by an infectious disease. The University of Maryland COVID Trends and Impact Survey provides an example of participatory surveillance that leveraged Facebook’s active user base to provide self-reported symptom and vaccination data in near real-time.

**Methods:**

Here, we develop a methodology for identifying changes in vaccine effectiveness and COVID-19 symptomatology using the University of Maryland COVID Trends and Impact Survey data from three middle-income countries (Guatemala, Mexico, and South Africa). We implement conditional logistic regression to develop estimates of vaccine effectiveness conditioned on the prevalence of various definitions of self-reported COVID-like illness in lieu of confirmed diagnostic test results.

**Results:**

We highlight a reduction in vaccine effectiveness during Omicron-dominated waves of infections when compared to periods dominated by the Delta variant (median change across COVID-like illness definitions: −0.40, IQR[−0.45, −0.35]. Further, we identify a shift in COVID-19 symptomatology towards upper respiratory type symptoms (i.e., cough and sore throat) during Omicron periods of infections. Stratifying COVID-like illness by the National Institutes of Health’s (NIH) description of mild and severe COVID-19 symptoms reveals a similar level of vaccine protection across different levels of COVID-19 severity during the Omicron period.

**Conclusions:**

Participatory surveillance data alongside methodologies described in this study are particularly useful for resource-constrained settings where diagnostic testing results may be delayed or limited.

## Plain language summary

Surveys that are sent out to users of social media can be used to supplement traditional methods to monitor the spread of infectious diseases. This has the potential to be particularly useful in areas where other data is unavailable, such as areas with less surveillance of infectious disease prevalence and access to infectious disease diagnostics. We used data from a survey available to users of the social media platform Facebook to collect information about any potential symptoms of COVID-19 infection and vaccines received during the COVID-19 pandemic. We found a potential reduction in vaccine effectiveness and change in symptoms when the Omicron variant was known to be circulating compared to the earlier Delta variant. This method could be adapted to monitor the spread of COVID-19 and other infectious diseases in the future, which might enable the impact of infectious diseases to be recognized more quickly.

## Abstract (web summary)

Varrelman et al. use participatory surveillance data to develop a method to identify changes in vaccine effectiveness and disease symptomatology. Using data from the COVID-19 pandemic, they identify a reduction in vaccine effectiveness during Omicron dominated waves of infections, and a shift towards upper respiratory type symptoms.

## Introduction

Timely identification of alterations in vaccine effectiveness (VE) with the emergence of novel COVID-19 variants, such as Omicron, is important for informing the global public health response. The attributable risk proportion of vaccine-preventable diseases is often estimated using relative risk measures obtained from cohort studies or odds ratios determined through case-control designs, which typically rely on gold-standard diagnostic testing^1,2^. These studies are conducted retrospectively, leading to a lag between variant emergence and VE estimates. In an effort to provide timely VE insights, monitoring systems have been developed that leverage digital health data^3,4^. However, even these real-time methodologies are bounded by some form of diagnostic testing data, whether it be self-reported or through other means of collection. While resource-rich locales across the world have managed to scale up diagnostic testing to inform pandemic response efforts, many low-and middle-income countries (LMICs) have struggled to establish widespread testing^5,6^, therefore limiting the applicability of current VE monitoring systems. Alternatively, digital health surveys of self-reported symptoms and vaccination status provide a data source that may be used in place of limited/delayed testing data^7–9^.

In this study, we use data from the University of Maryland Global COVID Trends and Impact Survey (UMD-CTIS) to develop a methodology to simultaneously characterize potential changes in VE and COVID-19 symptomatology for Delta and Omicron-dominated periods of infections. UMD-CTIS is a digital health survey that leveraged Facebook’s active user base, providing cross-sectional survey data in near real-time from 114 countries, starting in 2020 and ending in 2022. Our analyses utilize aggregate data from three MICs that were selected based on the quality of UMD-CTIS data and the presence of distinct Delta and Omicron periods of infections. The selected countries include Guatemala, Mexico, and South Africa. Our analyses of this data reveal reduced vaccine effectiveness against suspected COVID-19 infection during the Omicron period compared to Delta, as well as a shift towards more upper respiratory-type symptoms like cough and sore throat.

## Methods

### Syndromic surveillance data

The University of Maryland Global COVID Trends and Impact Survey (UMD-CTIS), in partnership with Facebook, is a cross-sectional survey that sampled Facebook’s active user base on a daily basis. Facebook users were presented an invitation at the top of their news feed, inviting them to participate in the survey. It is important to note that survey invitations did not include any type of incentive, and participation was driven purely by individuals’ willingness to contribute to digital health. If an individual decided to accept the invitation, they were navigated off of the Facebook platform to the digital health survey hosted by Qualtrics, with data collection being performed by the Joint Program in Survey Methodology at the University of Maryland. On the Qualtrics survey itself, respondents were shown the consent page explaining the purpose of the research to gain a better public understanding of where and how the coronavirus pandemic is spreading, that the survey would take 3–5 min, and that their responses would remain confidential and anonymous. After providing informed consent and confirmation of being at least 18 years of age, respondents could proceed with the survey. Survey respondents and non-respondents were entered back into the sampling pool after a duration of a few weeks or months, depending on the sample size for a given area. Survey data included self-reported information such as demographics, recent symptoms, and COVID-19 vaccination status. While Facebook acts as the survey sampling frame, the company cannot access individually identified respondent answers. Further, to work with these data, institutions must have a signed Data Use Agreement (data access and survey questions available https://covidmap.umd.edu)^7,10^, which our institution signed in order to access and analyze the UMD-CTIS data. Boston Children’s Hospital Institutional Review Board (P00023700) approved this study using UMD-CTIS data. Additional details on the survey design, methodology, and validation can be found in Astley et al. (2021)^7^.

To select the study locations, we began by focusing on countries that met three criteria: they are included in the UMD-CTIS sample, have encountered distinct waves of COVID-19 infections primarily driven by the Delta and Omicron variants, and are considered a low or middle-income country as described by the Organization for Economic Co-operation and Development (OECD). Next, we visualized the time-series symptom data and ruled out countries where the UMD-CTIS data was noticeably erratic.

Using peak detection (Python (3.8.2), scipy.signal.argrelextrema (1.7.1), order parameter = 70) for all CLI time series (April 2021–February 2022), we infer 2-week consensus variant periods prior to each peak, for Delta and Omicron, respectively, for Guatemala (peak date September 13, 2021 [survey No. 4137] and peak date February 2, 2022 [survey No. 2387]), South Africa (July 22, 2021 [survey No. 7371] and December 19, 2021 [survey No. 5320]), and Mexico (August 22, 2021 [survey No. 52775] and January 26, 2022 [survey No. 71990]), that coincided with >80% variant share per public reports^11^.

### Statistics and reproducibility

We utilize conditional logistic regression to estimate the attributable risk proportion (ARP) for illness in 2-dose vaccinated individuals (clogit function with method=’approximate’, R (4.1.1), survival library (3.2-13)). VE is given by VE = ARP ≈ 1−OR. We consider exposure as the vaccination status of a respondent (unvaccinated vs. 2-dose vaccinated), and the outcome as to whether a respondent reported CLI in past 14 days, with missing symptoms assumed absent. We also include strata for dichotomized age (>44 years), gender (male/female), and country of the survey respondent to limit potential confounding and differences in country-level sampling. Importantly, UMD-CTIS does not collect data on vaccine formulation. Consequently, we cannot definitively determine whether a single dose of any specific vaccine within our dataset consistently provides full protection, as seen with the Janssen COVID-19 vaccine formulation. Therefore, we have chosen not to include individuals who have received only one dose in this study. Age and gender were dichotomized in order to maintain sufficient sample sizes per stratum. We do not filter the individual vaccine effectiveness estimates by *p*-value, as we are interested in the group behavior of the CLI definitions and not the hypothesis of whether a single definition of CLI produces a statistically significant vaccine effectiveness estimate. Moreover, to maintain the same number of data points for each of our comparisons, we do not remove outlier data from the analyses in this study.

### Reporting summary

Further information on research design is available in the Nature Portfolio Reporting Summary linked to this article.

## Results

**Fig. 1:**
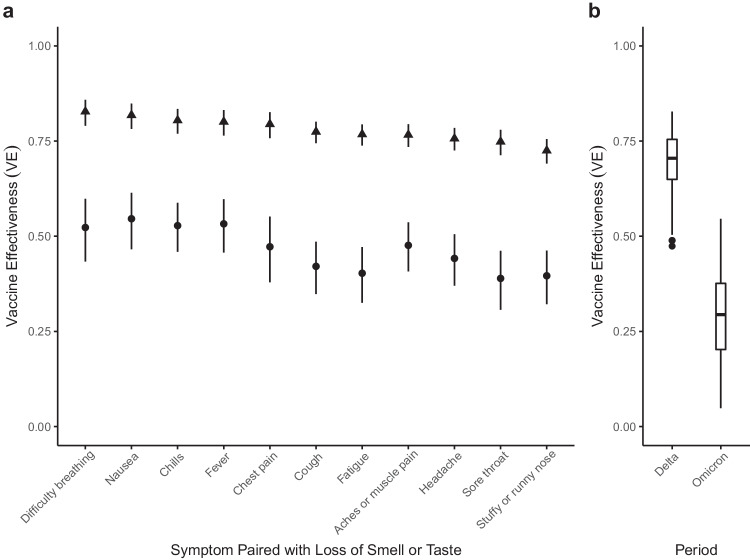
Vaccine effectiveness against COVID-like illness: Delta vs. Omicron. **a** VE estimates for symptoms paired with the loss of smell or taste for the Delta (triangle) and Omicron (circle) periods. 95% confidence intervals are calculated for each VE estimate, with Delta and Omicron period estimates derived from 64,283 and 79,697 survey responses, respectively. **b** Box and whisker plot of VE estimates across all 66 possible CLI defined by pairwise combinations of symptoms for Delta and Omicron periods. The box represents the interquartile range (IQR) of estimates, with the horizontal line inside the box indicating the median. The whiskers extend to the largest/smallest values up to 1.5 times the IQR. Outlier values are represented as points. The sample size for each VE estimate is consistent with the sample sizes described in panel (**a**).

**Fig. 2:**
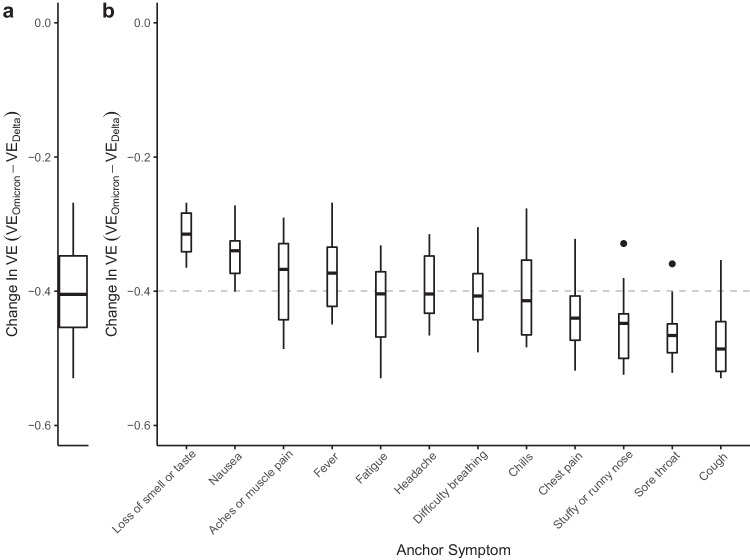
Change in vaccine effectiveness from Delta to Omicron. **a** Distribution of within-CLI change (VE_Omicron_−VE_Delta_) across all CLI definitions. **b** Distributions of VE_Omicron_−VE_Delta_ among CLI definitions within each anchor symptom. Each box-plot contains estimates for an anchor symptom paired with the 11 other symptoms. Box-plots are ordered according to the magnitude of the median change, with the median across all VE indicated by the gray dashed line. Each box represents the interquartile range (IQR) of estimates, with the horizontal line inside the box indicating the median. The whiskers extend to the largest/smallest values up to 1.5 times the IQR. Outlier values are represented as points. Each VE estimate from the Delta and Omicron periods is derived from 64,283 and 79,697 survey responses, respectively.

To estimate VE, we adapted case-control methods^1^ for prevalent COVID-like illness (CLI) as a proxy for confirmed COVID-19 cases. Therefore, our estimates of VE measure a vaccine’s ability to prevent suspected symptomatic infections defined by CLI. To allow for changes in variant-specific symptomatology, we iterate across all possible CLI defined by 66 pair-wise combinations of 12 self-reported symptoms (fever, cough, difficulty breathing, fatigue, stuffy or runny nose, aches or muscle pain, sore throat, chest pain, nausea, loss of smell or taste, headache, chills). We then cluster the vaccine effectiveness estimates according to a single symptom of interest and evaluate the median vaccine effectiveness across all CLI definitions in the cluster. As an example, using a COVID-19-specific symptom (loss of smell or taste) as an anchor symptom, we evaluate VE estimates for all CLI definitions inclusive of this symptom during Delta and Omicron waves of infections, resulting in VE estimates for 11 pairwise combinations of symptoms. Consistent with previous estimates of VE that used PCR test data as the outcome^2^, our analyses reveal a median VE_Delta_ of 0.77, IQR[0.76, 0.80] (Fig. 1a, triangle). In comparison, analyzing the data from the Omicron period reveals a median VE_Omicron_ of 0.47, IQR[0.41, 0.53] (Fig. 1a, circle). Further expanding the approach to all CLI definitions reveals a median VE_Delta_ of 0.71, IQR[0.65, 0.75] (Fig. 1b). In contrast, the VE_Omicron_ estimate is even lower (median 0.29, IQR[0.20, 0.38]). Notably, our findings align with those from a recent meta-analysis study focused on real-world vaccine effectiveness for fully vaccinated individuals. This study reported a VE of 70.9% (95% CI, 68.9–72.7) against Delta infections and a VE of 23.5% (95% CI, 17.0–29.5) against Omicron variant infections^12^. To understand how VE estimates for each CLI definition vary by wave, we take the difference between the two VE period estimates (VE_Omicron_−VE_Delta_) for each CLI definition. Doing so reveals a median within-CLI definition change of −0.40, IQR[−0.45, −0.35] (Fig. 2a), suggesting lower VE_Omicron_ regardless of the CLI definition that is used. Additionally, we find that the pattern of change in VE across CLI definitions is similar when evaluating individual country estimates (see Supplementary Fig. 1).

To identify potential alterations in COVID-19 symptomatology, we evaluate the change in VE estimates for CLI definitions with a single anchor symptom, like loss of smell and taste. We reason that if symptoms are similar across variants, the within-anchor median change in VE will be similar across anchor symptoms. Our analyses provide evidence for a potential change in COVID-19 symptomatology from the Delta period to the Omicron period, as we note that some symptoms have more or less decline in VE (Fig. 2b). Specifically, we find that CLI definitions that include loss of smell or taste have the smallest median change in VE (median: −0.31, IQR[−0.34, −0.28]), while definitions with the largest median change include a cough, or sore throat (cough median: −0.49, IQR[−0.52, −0.45]; sore throat median: −0.47, IQR[−0.49, −0.45]). The observed pattern of change in VE across anchor symptoms is similar when evaluating VE estimates from individual countries (see Supplementary Fig. 2), however, with increased uncertainty in estimates as measured by the span of anchor symptom distributions (see Supplementary Results). Similarly, a survey-based study that used PCR testing data as the outcome demonstrated a shift away from symptomatology that includes loss of smell or taste and towards upper-respiratory type symptoms (i.e., sore throat) during the Omicron period^13^. Furthermore, a study conducted in Jalisco, Mexico, analyzed reported symptoms for confirmed infections with wild-type SARS-CoV-2, Delta, and Omicron variants, revealing that Omicron infections were linked to a higher incidence of runny nose and sore throat, aligning with the findings of our country-level analysis for Mexico (see Supplementary Fig. 3)^14^. These results corroborate our overall findings, which also identified increased reporting of sore throat during a wave of COVID-19 infections dominated by the Omicron variant. Collectively, these findings suggest a shift in symptomatology associated with the Omicron variant towards more upper respiratory-type symptoms.

**Fig. 3:**
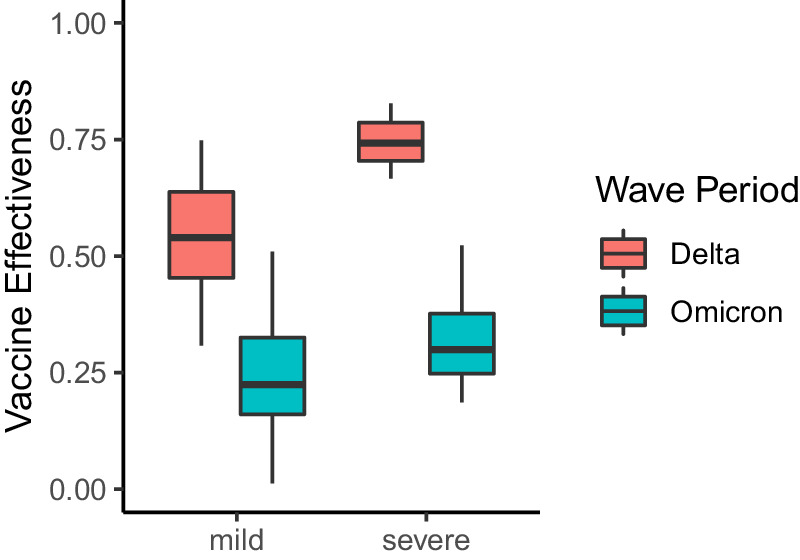
Vaccine effectiveness against mild and severe illness. VE estimates for pairwise combinations of symptoms that include a fever, cough, aches or muscle pain, sore throat, nausea, loss of smell or taste, or a headache in the absence of difficulty breathing or chest pain (mild illness), and pairwise combinations of symptoms that include difficulty breathing or chest pain (severe illness). Each box represents the interquartile range (IQR) of estimates, with the horizontal line inside the box indicating the median. The whiskers extend to the largest/smallest values up to 1.5 times the IQR. Outlier values are represented as points. Each VE estimate from the Delta and Omicron periods is derived from 64,283 and 79,697 survey responses, respectively.

In addition to providing insights into changes in COVID-19 symptomatology, the VE estimates also include information about a vaccine’s ability to protect against COVID-19 illness presenting at different levels of severity as defined by pairwise combinations of symptoms. Importantly, we do not have information about the true severity of each respondent’s reported illness, and we instead infer severity based on the presence and absence of key symptoms. For instance, all CLI definitions that include at least a fever, cough, aches or muscle pain, sore throat, nausea, loss of smell or taste, or a headache in the absence of difficulty breathing or chest pain are considered mild syndromes. However, according to the NIH, CLI definitions that include difficulty breathing or chest pain are considered more severe forms of illness^15^. To understand potential changes in VE against mild and severe COVID-19 syndromes, we partition our CLI-informed VE estimates according to the above classifications. As a result, we end up with 42 mild and 21 severe definitions of CLI. We find that severe definitions of illness were more protected than mild definitions during the Delta period (median severe VE: 0.74, IQR[0.70, 0.79], median mild VE: 0.54, IQR[0.45, 0.64]) (Fig. 3). However, protection against mild and severe illness was similar during Omicron (median severe VE: 0.30, IQR[0.25, 0.38], median mild VE: 0.22, IQR[0.16, 0.33]). Importantly, VE against severe illness may appear higher, as vaccines are producing milder illness when an individual is infected with COVID-19^16^, making it seem as if VE against mild illness is less effective. During the Delta wave of infections, we observed a total of 13,220 reports of mild illness and 5316 reports of severe illness. In contrast, during the Omicron wave of infections, there were 24,408 reports of mild illness and 10,234 reports of severe illness.

## Discussion

It is critical to note that our estimates of VE measure the preventable syndrome attributed to receiving 2-doses of vaccine and represent only one of many components that contribute to true vaccine effectiveness. For instance, we are unable to account for asymptomatic breakthrough infections, and we do not have information on natural immunity among the unvaccinated nor on vaccine formulation or timing for the vaccinated. Therefore, we do not have enough information to distill whether changes in VE are caused by waning vaccine immunity, or increased penetration of an emerging variant. To this end, we would suggest that future digital health surveys include information on vaccine formulation, the general timing of vaccination, as well as information on booster doses that have been administered. While quickly adapting a digital health survey is a monumental task, it would enhance the capabilities of methods such as those described in this study. Furthermore, our VE estimates are solely derived from self-reported survey data and are thus vulnerable to a range of biases^17^. For instance, self-report bias is likely influenced by the perception around COVID-19 vaccination at a given time for a given locale. Even so, a U.S.-based survey that incorporated viral testing demonstrated that self-reported vaccination is a strong predictor for true vaccination status^18^, thus providing support for self-reported measures. Further, our estimates rely on the assumption that the range of self-reported CLI definitions defined in this study is a valid proxy for incident COVID-19 infection. Consequently, our VE estimates may be an underestimation if CLI is capturing non-COVID illness. We limit this assumption by selecting time periods reflective of when COVID-19 is circulating within the unvaccinated population of survey respondents for each country.

Although the assumptions mentioned above limit the interpretation of our VE estimates, the methodology still demonstrates notable strengths that should not be discounted. For example, simple surveys that collect self-reported symptoms and vaccination status can be collected rapidly and at a fraction of the cost of traditional surveillance measures^19^. Moreover, while we performed the retrospective analysis with knowledge of specific COVID-19 variants, CLI-informed VE estimates can be derived during suspected variant spread, with careful contextualization of a country’s epidemiological situation (i.e., absence of co-circulating pathogens and sufficient geographic coverage of surveys). In the case of UMD-CTIS, there was a two-week delay between survey completion and its availability for our modeling, allowing us to use it as a valuable near-real-time dataset for VE analyses. It is critical to note that UMD-CTIS collected a substantial number of survey samples from numerous countries, enabling meaningful insights into COVID-19. However, some countries within the UMD-CTIS sample exhibited noisy data, characterized by high variability in the number of reported CLI instances between time steps, which limited the utility of these specific datasets. While UMD-CTIS has yielded valuable data from a wide range of countries, it’s important to acknowledge that the determination of survey sampling intensity, size, and other attributes of sampling can impact the reliability and applicability of findings. To truly understand the minimum number of samples required for robust statistical analyses, further research, and investigation into these sampling parameters are essential. Such efforts will not only enhance the effectiveness of syndromic surveillance but also contribute to more accurate and comprehensive insights into COVID-19 dynamics.

Historically, understanding the impact of infectious diseases, including the effectiveness of vaccination, has relied on detailed clinical data, often gathered through sentinel surveillance networks^20^. For example, the CDC’s U.S. Outpatient Influenza-like Illness Surveillance Network (ILINet) provides information about symptom prevalence for suspected flu cases across the United States over time. While an invaluable resource, ILINet is limited to individuals seeking medical care due to its reliance on sentinel providers for data collection. Therefore, individuals who lack access to such sentinel providers or those who do not seek care will not be represented in these data. Consequently, epidemiological parameters derived from these data may not be entirely representative of the population of interest. Participatory digital surveillance systems like Flu Near You, the ZOE App, and UMD-CTIS enable broader symptom tracking by collecting data directly from the public^3,21^. These community-based data sources can provide complementary signals to those derived through clinical data-dependent systems like ILINet^22^. Our analysis of self-reported symptoms from UMD-CTIS demonstrates how digital health data can also be rapidly utilized to infer symptomatic shifts across populations, with the advantage of timeliness and scope beyond only those seeking care. While this application does not provide the same level of clinical confirmation as traditional studies, combining evidence from both clinical and digital participatory data sources allows for earlier response guidance while gold-standard data are collected. For instance, applying our methodology of detecting potential changes in symptomatology could help direct early public health mitigation strategies.

The COVID-19 pandemic exposed vulnerabilities in health infrastructure, particularly for LMICs that struggled to establish testing facilities^8^, needed to support real-time epidemiological parameter estimation that depends on diagnostic testing results. Leveraging the power of global participatory epidemiology in the form of digital health surveys^23^ has the potential to supplement these critical testing gaps. Thus, our methods of using self-reported symptom data to understand VE and changes in symptomatology is a powerful rapid response tool, that can provide the medical community with timely insights into emerging variants. Due to our agnostic approach in defining a syndrome (i.e., all pairwise symptoms), the utility of our methods goes beyond COVID-19 and can be applied to other upper-respiratory illnesses and/or locations to support response to emerging threats.

### Supplementary information

Supplemental Material

Description of Additional Supplementary Files

Supplemental Data 1

Supplemental Data 2

Reporting Summary

## Data Availability

To access the raw data used in this manuscript, a request must be submitted to the Facebook Data for Good website: https://dataforgood.facebook.com/dfg/docs/covid-19-trends-and-impact-survey-request-for-data-access. The Global UMD-CTIS Open Data API, Microdata Repository, and contingency tables are available from The University of Maryland Social Data Science Center Global COVID-19 Trends and Impact Survey website (https://covidmap.umd.edu). The results of the conditional logistic regression can be found in Supplemental Data 1 and Supplemental Data 2.

## Code availability

The R and Python code used to perform the analyses in this study is available at 10.5281/zenodo.10775701^24^.

## References

[1] Collie S, Champion J, Moultrie H, Bekker L-G, Gray G (2022). Effectiveness of bnt162b2 vaccine against omicron variant in South Africa. N. Engl. J. Med..

[2] Andrews N (2022). Covid-19 vaccine effectiveness against the omicron (b.1.1.529) variant. N. Engl. J. Med..

[3] Menni, C. et al. Covid-19 vaccine waning and effectiveness and side-effects of boosters: a prospective community study from the Zoe Covid study. *Lancet Infect. Dis.* **22**, 1002–1010 (2022). 10.1016/S1473-3099(22)00146-3 PMC8993156 35405090

[4] Bitzegeio, J., Hemmers, L., Bartel, A. & Werber, D. Timely monitoring COVID-19 vaccine protection, Berlin, Germany, April 15 to December 15, 2021. *Int. J. Public Health* **67**, 1604633 (2022). 10.3389/ijph.2022.1604633 PMC8977412 35387147

[5] Batista C (2022). The silent and dangerous inequity around access to COVID-19 testing: a call to action. EClinicalMedicine.

[6] Aziz AB (2020). Integrated control of COVID-19 in resource-poor countries. Int. J. Infect. Dis..

[7] Astley, C. M. et al. Global monitoring of the impact of the COVID-19 pandemic through online surveys sampled from the Facebook user base. *Proc. Natl Acad. Sci. USA* **118**, https://www.pnas.org/content/118/51/e2111455118 (2021). 10.1073/pnas.2111455118 PMC8713788 34903657

[8] Fulcher IR (2021). Syndromic surveillance using monthly aggregate health systems information data: methods with application to COVID-19 in Liberia. Int. J. Epidemiol..

[9] Eames K (2012). Rapid assessment of influenza vaccine effectiveness: analysis of an internet-based cohort. Epidemiol. Infect..

[10] Kreuter F (2020). Partnering with a global platform to inform research and public policy making. Survey Res. Methods.

[11] Hodcroft, E. B. Covariants: SARS-CoV-2 Mutations and Variants of Interest (accessed 30 March 2022). *CoVariants* https://covariants.org/ (2022).

[12] Zeng B, Gao L, Zhou Q, Yu K, Sun F (2022). Effectiveness of COVID-19 vaccines against SARS-CoV-2 variants of concern: a systematic review and meta-analysis. BMC Med..

[13] Vihta, K.-D. et al. Omicron-associated changes in severe acute respiratory syndrome coronavirus 2 (sars-cov-2) symptoms in the United Kingdom. *Clin. Infect. Dis.* **76**, e133–e141 (2022). 10.1093/cid/ciac613 PMC9384604 35917440

[14] Peña Rodríguez, M. et al. Prevalence of symptoms, comorbidities, and reinfections in individuals infected with wild-type sars-cov-2, delta, or omicron variants: a comparative study in western Mexico. *Front. Public Health* **11**, 1149795 (2023). 10.3389/fpubh.2023.1149795 PMC10174068 37181688

[15] National Institutes of Health. Clinical Spectrum of SARS-COV-2 infection (accessed 12 June 2022). *National Institutes of Health* https://www.covid19treatmentguidelines.nih.gov/overview/clinical-spectrum/ (2022).

[16] Centers for Disease Control and Prevention. Covid-19 After Vaccination: Possible Breakthrough Infection (accessed 12 June 2022). *Centers for Disease Control and Prevention* https://www.cdc.gov/coronavirus/2019-ncov/vaccines/effectiveness/why-measure-effectiveness/breakthrough-cases.html (2022).

[17] Bradley, V. C. et al. Unrepresentative big surveys significantly overestimated us vaccine uptake. *Nature*10.1038/s41586-021-04198-4(2021). 10.1038/s41586-021-04198-4 PMC8653636 34880504

[18] Siegler AJ (2021). Trajectory of COVID-19 vaccine hesitancy over time and association of initial vaccine hesitancy with subsequent vaccination. JAMA Netw. Open.

[19] Aiello AE, Renson A, Zivich P (2020). Social media-and Internet-based disease surveillance for public health. Annu. Rev. Public Health.

[20] Torner N (2014). Influenza vaccine effectiveness assessment through sentinel virological data in three post-pandemic seasons. Hum. Vaccines Immunother..

[21] Chunara, R., Aman, S., Smolinski, M. S. & Brownstein, J. S. Flu near you: an online self-reported influenza surveillance system in the USA. *Online J. Public Health Inform.* **5**, https://api.semanticscholar.org/CorpusID:31097654 (2013).

[22] Wójcik O, Brownstein J, Chunara R, Johansson M (2014). Public health for the people: participatory infectious disease surveillance in the digital age. Emerg. Themes Epidemiol..

[23] Chan AT, Brownstein JS (2020). Putting the public back in public health—surveying symptoms of COVID-19. N. Engl. J. Med..

[24] Varrelman, T. *Code from: Vaccine Effectiveness Against Emerging COVID-19 Variants using Digital Health Data*10.5281/zenodo.1077570110.5281/zenodo.10775701 (2024). 10.1038/s43856-024-00508-9 PMC11074297 38710936

