# Supplementary material for: Vaccine effectiveness against emerging COVID-19 variants using digital health data
Source: Commun Med (Lond). 2024 May 6;4:81. doi: 10.1038/s43856-024-00508-9 (PMC11074297; doi:10.1038/s43856-024-00508-9)
Supplement: Supplementary file 1 — Supplemental Material [file 43856_2024_508_MOESM1_ESM.pdf]

Vaccine Effectiveness Against Emerging COVID-19 Variants Using Digital Health Data

Tanner J. Varrelman[1,*], Benjamin Rader[1,2], Christopher Remmel[1], Gaurav Tuli[1], Aimee R. Han[1], Christina M. Astley[1,3,4,5], John S. Brownstein[1,4]

[1]Computational Epidemiology Lab, Boston Children's Hospital, Boston, MA 02115, United States;
[2]Department of Epidemiology, Boston University, Boston, MA 02118, United States;
[3]Division of Endocrinology, Boston Children's Hospital, Boston, MA 02115, United States;
[4]Harvard Medical School, Boston, MA 02115, United States;
[5]Broad Institute of Harvard and MIT, Cambridge, MA 02142, United States;
* Corresponding Author: tannervarrelman@gmail.com

Supplementary Information

Supplementary Results

Main text analyses rely on the aggregate of country data to provide a robust sample size for estimating vaccine effectiveness (VE). While main text analyses provide general insights into potential changes in VE, these estimates do not provide the necessary information to make country level comparisons. Here, we expand the analyses described in the main text to develop country specific COVID-like illness (CLI) informed VE estimates for Mexico, South Africa, and Guatemala. Doing so, we find that VE estimates declined from the

Delta to Omicron period across all three locales. Mexico experienced a median within-CLI change of the largest magnitude -0.43, IQR[-0.48, -0.38], followed by South Africa (median change: -0.31, IQR[-0.37, -0.25]), and finally Guatemala (median change: -0.21, IQR[-0.28,-0.14]) (Supplementary Figure 1).

To better understand the observed variation in VE reduction across countries, we evaluate the distribution of within-CLI change across anchor symptoms (Supplementary Figure 2). We find that generally, the pattern of change across anchor symptoms is similar across the study regions (Supplementary Figure 3). Specifically, loss of smell/taste remains an anchor symptom that experiences one of the smallest median reductions across each country, while CLI definitions that include a cough, sore throat, or stuffy/runny nose experience larger median reductions. However, Supplementary Figure 2 also highlights the considerable variation in the span of anchor symptom distributions across countries. We find that the greatest variation in anchor symptom distributions occurs in Guatemala, the country with the smallest sample size in our study. It is unclear whether the observed differences in VE estimates across countries are due to biological mechanisms (i.e. different vaccine formulations), or differences in sample size.

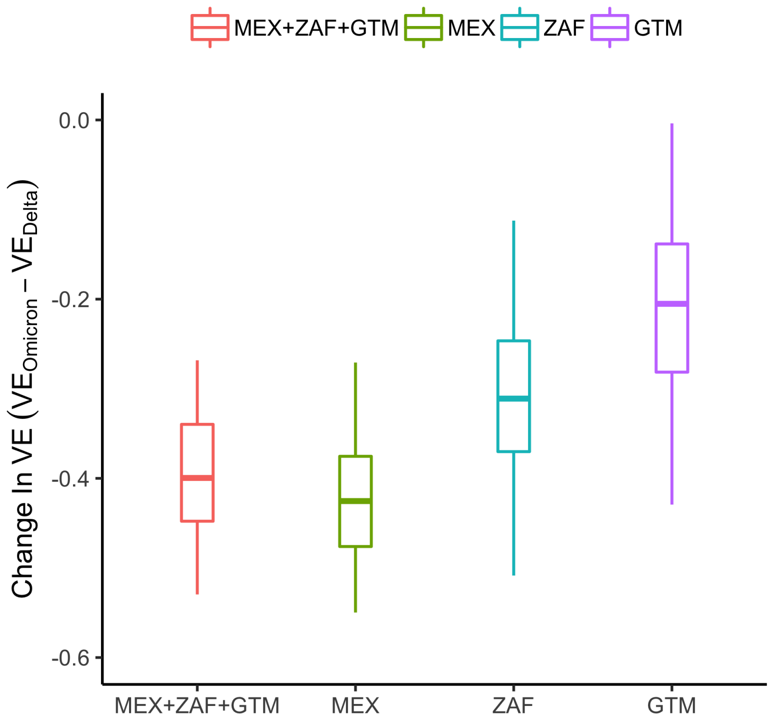

Supplementary Figure 1: Distribution of within-CLI change ($VE_{Omicron}$-VE_{Delta}) across all CLI definitions, for the aggregate of countries (red), Mexico (MEX, green), South Africa (ZAF, blue), and Guatemala (GTM, purple).

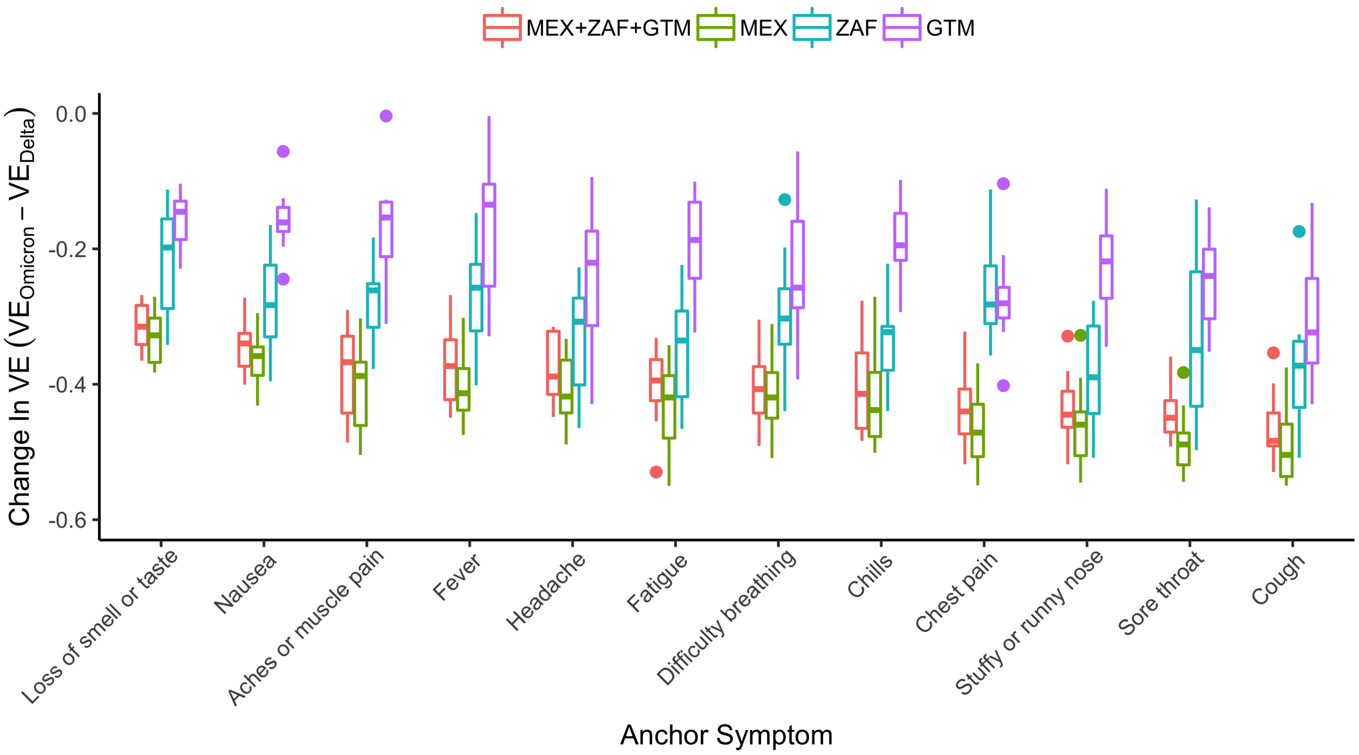

Supplementary Figure 2: Distributions of $VE_{Omicron}$-VE_{Delta} among CLI definitions within each anchor symptom, for the aggregate of countries (red), Mexico (MEX, green), South Africa (ZAF, blue), and Guatemala (GTM, purple). Each box-plot contains estimates for an anchor symptom paired with the 11 other symptoms.

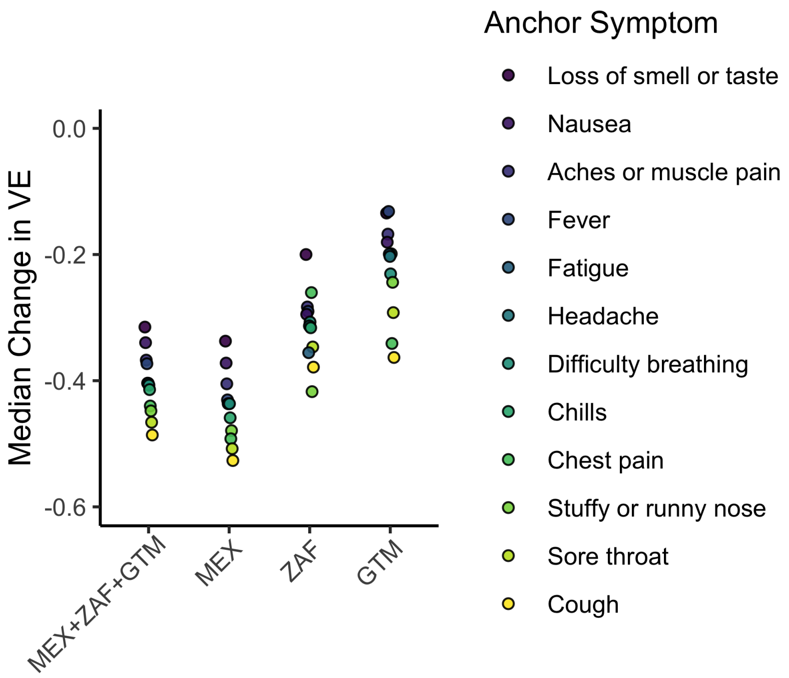

Supplementary Figure 3: Median change among CLI definitions within each anchor symptom for the aggregate of counties (MEX+ZAF+GTM), Mexico (MEX), South Africa (ZAF), and Guatemala (GTM).
