# Supplementary material for: Vaccine effectiveness against emerging COVID-19 variants using digital health data
Source: Commun Med (Lond). 2024 May 6;4:81. doi: 10.1038/s43856-024-00508-9 (PMC11074297; doi:10.1038/s43856-024-00508-9)
Supplement: Supplementary file 2 — Description of Additional Supplementary Files [file 43856_2024_508_MOESM2_ESM.pdf]

Description of Additional Supplementary Files

File name: Supplemental Data 1
Description: This file contains the regression results used to create Figures 1 and 2. This dataset contains critical information such as the vaccine effectiveness estimate, confidence intervals, and p-values.

File name: Supplemental Data 2
Description: This file contains the regression results used to create Figure 3. This dataset contains critical information such as the vaccine effectiveness estimate, confidence intervals, and p-values.
