# Supplementary material for: Vaccine effectiveness against emerging COVID-19 variants using digital health data
Source: Commun Med (Lond). 2024 May 6;4:81. doi: 10.1038/s43856-024-00508-9 (PMC11074297; doi:10.1038/s43856-024-00508-9)
Supplement: Supplementary file 5 — Reporting Summary [file 43856_2024_508_MOESM5_ESM.pdf]

Corresponding author(s): Tanner J. Varrelman

Last updated by author(s): Mar 3, 2024

Reporting Summary

Nature Portfolio wishes to improve the reproducibility of the work that we publish. This form provides structure for consistency and transparency in reporting. For further information on Nature Portfolio policies, see our Editorial Policies and the Editorial Policy Checklist.

Statistics

For all statistical analyses, confirm that the following items are present in the figure legend, table legend, main text, or Methods section.

n/a	Confirmed	
☐	☒	The exact sample size (*n*) for each experimental group/condition, given as a discrete number and unit of measurement
☐	☒	A statement on whether measurements were taken from distinct samples or whether the same sample was measured repeatedly
☐	☒	The statistical test(s) used AND whether they are one- or two-sided<br>*Only common tests should be described solely by name; describe more complex techniques in the Methods section.*
☐	☒	A description of all covariates tested
☐	☒	A description of any assumptions or corrections, such as tests of normality and adjustment for multiple comparisons
☐	☒	A full description of the statistical parameters including central tendency (e.g. means) or other basic estimates (e.g. regression coefficient) AND variation (e.g. standard deviation) or associated estimates of uncertainty (e.g. confidence intervals)
☒	☐	For null hypothesis testing, the test statistic (e.g. *F*, *t*, *r*) with confidence intervals, effect sizes, degrees of freedom and *P* value noted<br>*Give P values as exact values whenever suitable.*
☒	☐	For Bayesian analysis, information on the choice of priors and Markov chain Monte Carlo settings
☒	☐	For hierarchical and complex designs, identification of the appropriate level for tests and full reporting of outcomes
☒	☐	Estimates of effect sizes (e.g. Cohen's *d*, Pearson's *r*), indicating how they were calculated

Our web collection on statistics for biologists contains articles on many of the points above.

Software and code

Policy information about availability of computer code

Data collection	No software was used.
Data analysis	Analyses in this study are reliant on Python (3.8.2), Python package scipy.signal.agrelextrema (1.7.1), R (4.1.1), and the R Survival library (3.2-13).

For manuscripts utilizing custom algorithms or software that are central to the research but not yet described in published literature, software must be made available to editors and reviewers. We strongly encourage code deposition in a community repository (e.g. GitHub). See the Nature Portfolio guidelines for submitting code & software for further information.

Data

Policy information about availability of data

All manuscripts must include a data availability statement. This statement should provide the following information, where applicable:

- Accession codes, unique identifiers, or web links for publicly available datasets
- A description of any restrictions on data availability
- For clinical datasets or third party data, please ensure that the statement adheres to our policy

To access the raw data used in this manuscript, a request must be submitted to the Facebook Data for Good website: https://dataforgood.facebook.com/dfg/docs/covid-19-trends-and-impact-survey-request-for-data-access. The Global UMD-CTIS Open Data API, Microdata Repository, and contingency tables are available from The University of Maryland Social Data Science Center Global COVID-19 Trends and Impact Survey website (https://covidmap.umd.edu).

Human research participants

Policy information about studies involving human research participants and Sex and Gender in Research.

Reporting on sex and gender	*Use the terms sex (biological attribute) and gender (shaped by social and cultural circumstances) carefully in order to avoid confusing both terms. Indicate if findings apply to only one sex or gender; describe whether sex and gender were considered in study design whether sex and/or gender was determined based on self-reporting or assigned and methods used. Provide in the source data disaggregated sex and gender data where this information has been collected, and consent has been obtained for sharing of individual-level data; provide overall numbers in this Reporting Summary. Please state if this information has not been collected. Report sex- and gender-based analyses where performed, justify reasons for lack of sex- and gender-based analysis.*
Population characteristics	See above.
Recruitment	See sampling strategy above.
Ethics oversight	Boston Children's Hospital Institutional Review Board (P00023700) approved this study using UMD-CTIS data.

Note that full information on the approval of the study protocol must also be provided in the manuscript.

Field-specific reporting

Please select the one below that is the best fit for your research. If you are not sure, read the appropriate sections before making your selection.

☐ Life sciences ☒ Behavioural & social sciences ☐ Ecological, evolutionary & environmental sciences

For a reference copy of the document with all sections, see nature.com/documents/nr-reporting-summary-flat.pdf

Behavioural & social sciences study design

All studies must disclose on these points even when the disclosure is negative.

Study description	In this study, we utilized qualitative survey data from the University of Maryland COVID Trends and Impact Survey (UMD-CTIS) to develop estimates of vaccine effectiveness, informed by COVID-like illness.
Research sample	In this study we relied on UMD-CTIS data from survey respondents living in Guatemala, South Africa, and Mexico. Our study leveraged information about the survey respondents gender (male, female), dichotomized age (>44 years), vaccination status (unvaccinated, 2-dose vaccinated), and whether or not the respondent experienced COVID-like illness in the past 24 hours. We chose geographic regions that experienced distinct waves of Delta and Omicron infections.
Sampling strategy	Per the UMD-CTIS website (https://covidmap.umd.edu): "The surveys are daily repeated cross-sections, with similar user characteristics across days. The Facebook app invites a new sample of adult users to take the survey each day. Sampled users see an invitation at the top of their Facebook News Feed to an optional, off-Facebook survey. In order to provide geographic coverage, we use stratified random sampling using administrative boundaries within countries or territories. And, in order to reduce survey fatigue over the course of field collection, we employ differential sampling probabilities across these administrative boundaries. Sampled users may be invited to take the survey again in either a few weeks or months, depending on the population density of their area."
Data collection	Digital surveys were completed using a smartphone or computer.
Timing	UMD-CTIS started data collection in April 2020, with continuous collection
Data exclusions	Because we defined model strata that included the age and gender of survey respondents, we excluded data points that were missing either of these fields.
Non-participation	This information is not made available for the consumers of the UMD-CTIS data.
Randomization	Survey respondents were not allocated into random experimental groups.

Reporting for specific materials, systems and methods

We require information from authors about some types of materials, experimental systems and methods used in many studies. Here, indicate whether each material, system or method listed is relevant to your study. If you are not sure if a list item applies to your research, read the appropriate section before selecting a response.

Materials & experimental systems

n/a	Involved in the study
☒	☐ Antibodies
☒	☐ Eukaryotic cell lines
☒	☐ Palaeontology and archaeology
☒	☐ Animals and other organisms
☒	☐ Clinical data
☒	☐ Dual use research of concern

Methods

n/a	Involved in the study
☒	☐ ChIP-seq
☒	☐ Flow cytometry
☒	☐ MRI-based neuroimaging
